# Comparative analysis of the GATA transcription factors in seven *Ipomoea* species

**DOI:** 10.3389/fpls.2025.1714791

**Published:** 2025-11-19

**Authors:** Zengzhi Si, Jiuting Guo, Zhixin Ji, Fengrui Men, Weicao Wang

**Affiliations:** Hebei Key Laboratory of Crop Stress Biology, Hebei Normal University of Science and Technology, Qinhuangdao, Hebei, China

**Keywords:** *Ipomoea* species, *GATA* genes, phylogenetic analysis, chromosome location, duplication analysis, cis-regulatory elements, expression patterns, stresses response

## Abstract

The GATA transcription factors regulate plant growth, development, and stress responses, but our knowledge of their functions in sweetpotato and related *Ipomoea* species remains limited. Through analytical methods of bioinformatics, this study identified 410 *GATA* genes across seven sequenced *Ipomoea* species: sweetpotato (158), *I. trifida* (54), *I. triloba* (62), *I. nil* (39), *I. purpurea* (32), *I. cairica* (32), and *I. aquatica* (33). Phylogenetic analysis revealed that these *GATA* genes clustered into four distinct subfamilies (I-IV). Chromosomal mapping showed an uneven distribution pattern, with complete absence of *GATA* genes on certain chromosomes in each species. Duplication analysis indicated differential expansion mechanisms: tandem duplications primarily drove *GATA* gene expansion in *I. triloba*, *I. trifida*, and *I. nil*, whereas segmental duplications were predominant in sweetpotato and *I. cairica*. Promoter analysis identified multiple stress-responsive *cis*-regulatory elements, including ABRE, ARE, CGTCA-motif, GC-motif, LTR, MBS, TCA-element, TC-rich repeats, and TGACG-motif. Expression profiling under various stresses (salt, drought, *Ceratocystis fimbriata* and *Ditylenchus destructor)* detected 29–60 differentially expressed *GATA* genes (DEGs). Three representative DEGs (*IbGATA33*, *IbGATA38*, and *IbGATA126*) were validated by qRT-PCR, with results corroborating the transcriptome data. This study may contribute to further understanding of the evolution and function of *GATA* genes among the *Ipomoea* species, including sweetpotato.

## 1 Introduction

Plants undergo intricate biological processes throughout their life cycle, including seed germination, vegetative growth, reproductive development, and responses to stresses. These processes are precisely regulated by multi-layered molecular networks, in which transcription factors serve as central regulators of gene expression by specifically binding to promoter regions of target genes, thereby activating or suppressing downstream functional gene expression (Todeschini et al., 2014). Among transcriptional regulators, GATA-family transcription factors have been increasingly recognized as crucial molecular players that orchestrate diverse physiological processes in plants, including but not limited to growth regulation, developmental programming, and sophisticated stress adaptation responses (Schwechheimer et al., 2022).

GATA transcription factors are widely distributed across eukaryotes, including animals, plants, and fungi, and play pivotal roles in critical physiological processes (Zhao et al., 2023). In 1988, Evans et al. first identified GATA factor in chicken erythrocytes, demonstrating its role in hematopoiesis through regulation of globin gene expression (Evans et al., 1988). Subsequently, the first plant *GATA* gene *NTL1*, involved in nitrogen metabolism, was cloned from tobacco (Daniel-Vedele and Caboche, 1993). Thereafter, extensive research has revealed the crucial involvement of GATA transcription factors in modulating plant growth, development, and abiotic stress tolerance mechanisms.

In *Arabidopsis thaliana*, GATA2 transcription factor orchestrates photomorphogenesis and serves as a critical component in light signaling pathways (Luo et al., 2010); ectopic overexpression of *AtGNC* or *AtCGA1* significantly enhances chloroplast biogenesis in both hypocotyl cortex and root pericycle cells of *Arabidopsis* (Zhang et al., 2020). In rice, NECK LEAF 1, a GATA type transcription factor, modulates organogenesis by regulating the expression of multiple regulatory genes during reproductive development (Wang et al., 2009); *OsGATA12* overexpression restricts leaf and tiller development, thereby affecting yield-related characteristics (Lu et al., 2017); and *OsGATA7* coordinates brassinosteroid-mediated architectural modifications that influence both grain morphology and yield parameters (Zhang et al., 2018). In wheat, functional characterization reveals that TaGATA1 positively regulates wheat resistance to *Rhizoctonia cerealis*, as evidenced by enhanced disease tolerance in overexpression lines and increased susceptibility in silenced plants (Wei et al., 2023). Additionally, heterologous expression of soybean *GmGATA58* in *Arabidopsis* enhances leaf chlorophyll accumulation while simultaneously inhibiting plant growth and reducing yield (Zhang et al., 2020); transgenic overexpression of *SlGATA17* in tomato enhances drought tolerance through modulation of phenylpropanoid biosynthesis pathway activity (Zhao et al., 2021b); *IbGATA24* overexpression in sweetpotato plants establishes a molecular module with COP9-5a to coordinately enhance abiotic stress tolerance against both water deficit and high salinity conditions (Zhu et al., 2022).

GATA transcription factors derive their nomenclature from their conserved ability to recognize and bind the canonical (T/A)GATA(A/G) consensus sequence within promoter regions of target genes (Omichinski et al., 1993). These transcription factors contain a characteristic type-IV zinc finger domain featuring the conserved CX_2_CX_17−20_CX_2_C motif, with an adjacent basic region that mediates DNA binding (Reyes et al., 2004). A conserved GATA-type zinc finger domain containing 17–18 residues in the binding loop is characteristic of animal and fungal GATA transcription factors, whereas plant GATA factors typically exhibit an extended loop of 17–20 residues (Reyes et al., 2004; Park et al., 2006; Gupta et al., 2017). Systematic analysis of conserved structural motifs and phylogenetic relationships divides plant GATA transcription factors into four evolutionarily distinct classes (Reyes et al., 2004).

Given the importance of the GATA transcription factors in plants, they have been characterized across diverse plant lineages, including 29 in *Arabidopsis* (Reyes et al., 2004), 28 in rice (Reyes et al., 2004), 79 in wheat (Feng et al., 2022), 88 in tetraploid potato (Zhang et al., 2024), 38 in poplar (Zhao et al., 2023), 24 in melon (Zheng et al., 2024b), 24 in onion (Bose et al., 2025), and so on. As discussed above, the number of *GATA* genes varied greatly in the genomes of different species. Additionally, cross-species comparative genomics approaches have been conducted, for instance, in five Solanaceae species (*Lycium barbarum*, *Solanum lycopersicum*, *Capsicum annuum*, *Solanum tuberosum*, and *Solanum melongena*) (Zhang et al., 2023), in seven Orchidaceae species (*Phalaenopsis equestris*, *Cymbidium goeringii*, *C. ensifolium*, *Dendrobium catenatum*, *D. chrysotoxum*, *D. nobile*, *and Gastrodia elata*) (Zheng et al., 2024c), and in seven *Populus* species (*P. tremuloides*, *P. tremula*, *P. tremula x alba*, *P. pruinosa*, *P. euphratica*, *P. trichocarpa* and *P. deltoides*) (Kim et al., 2021b). These investigations systematically characterize *GATA* gene functions across phylogenetically diverse species, establishing both fundamental evolutionary insights and practical genetic reservoirs for plant enhancement programs targeting yield improvement and environmental stress adaptation.

*Ipomoea*, the most species-rich genus in the Convolvulaceae family, comprises approximately 600–700 species with a cosmopolitan distribution (Austin et al., 2015). This taxon holds significant economic value across agricultural, pastoral, and industrial sectors (Liu, 2011). Taking sweetpotato as an example, as the seventh most important crop worldwide, it serves as both an indispensable food and feed crop and a primary industrial raw material for energy production (Liu, 2011; 2017). Despite the importance of GATA transcription factors and *Ipomoea* species, comparative analyses of these factors across Ipomoea species remain limited.

This study conducted a genome-wide comparative analysis of the *GATA* gene family in seven *Ipomoea* species. A total of 158, 54, 62, 39, 32, 32 and 33 *GATA* genes were identified from sweetpotato (*I. batatas*), *I. trifida*, *I. triloba*, *I. nil*, *I. purpurea*, *I. cairica* and *I. aquatica*, respectively. An extensive characterization of the *GATA* gene family was performed, including analyses of gene structure, conserved protein motifs, phylogenetic relationships, chromosomal localization, gene duplication events, syntenic relationships, and evolutionary selection pressure (Ka/Ks ratios). Subsequently, tissue-specific and stress-responsive RNA-seq datasets were employed to analyze the expression patterns of these genes. The results revealed that 98 differentially expressed genes (DEGs) in sweetpotato, and three of them were subsequently validated through quantitative reverse-transcription PCR (qRT-PCR). This study provides fundamental genomic insights into *Ipomoea GATA* gene functions, establishing a crucial knowledge base for sequential investigations of their biological roles while facilitating molecular breeding applications in sweetpotato improvement programs.

## 2 Results

### 2.1 Identification of the *GATA* genes in the seven *Ipomoea* species

Genome-wide analysis identified 410 *GATA* genes across seven *Ipomoea* species: 158 in sweetpotato (*IbGATA1-158*), 54 in *I. trifida* (*ItfGATA1-54*), 62 in *I. triloba* (*ItbGATA1-62*), 39 in *I. nil* (*InGATA1-39*), 32 each in *I. purpurea* (*IpGATA1-32*) and *I. cairica* (*IcGATA1-32*), and 33 in *I. aquatica* (*IaGATA1-33*), representing 0.09%, 0.12%, 0.13%, 0.09%, 0.10%, 0.08%, and 0.06% of their respective genomes (**Supplementary File 1: Supplementary Table S1**). Comparative analysis revealed that the average protein length was 301.26 amino acids (range: 95–992 aa), with *I. cairica* showing the longest average (341.44 aa; range: 143–851 aa), followed by *I. purpurea* (336.06 aa; 151–930 aa), *I. aquatica* (327.79 aa; 148–543 aa), *I. trifida* (310.89 aa; 133–540 aa), *I. triloba* (302.35 aa; 95–540 aa), *I. nil* (290.97 aa; 139–535 aa), and sweetpotato (279.36 aa; 134–992 aa). Exon analysis showed an average of 4.16 exons per gene (range: 1-21), with *I. cairica* again having the highest average (4.62), followed by *I. purpurea* (4.56), *I. triloba* (4.53), *I. nil* (4.41), *I. trifida* (4.22), *I. aquatica* (4.09), and sweetpotato (3.77) (**Supplementary File 1: Supplementary Table S1**). The physicochemical characterization of *Ipomoea* GATA transcription factors revealed an average molecular weight of 32,968.81 Da (range: 10,952.43-107,174.11 Da), with isoelectric points averaging 7.60 (range: 4.67-10.80) and hydropathicity values averaging -0.64 (range: -1.20 to -0.24). Subcellular localization predictions indicated predominant nuclear localization (329 proteins, 80.24%), with minority distributions in chloroplasts (64, 15.60%), cytoplasm (9, 2.20%), and mitochondria (4, 0.98%). Singular instances were predicted for cytoplasmplasm, endoplasmic reticulum, extracellular space, and peroxisomes (**Supplementary File 1: Supplementary Table S1**).

### 2.2 Phylogenetic analysis of the *Ipomoea GATA* genes

**Figure 1:**
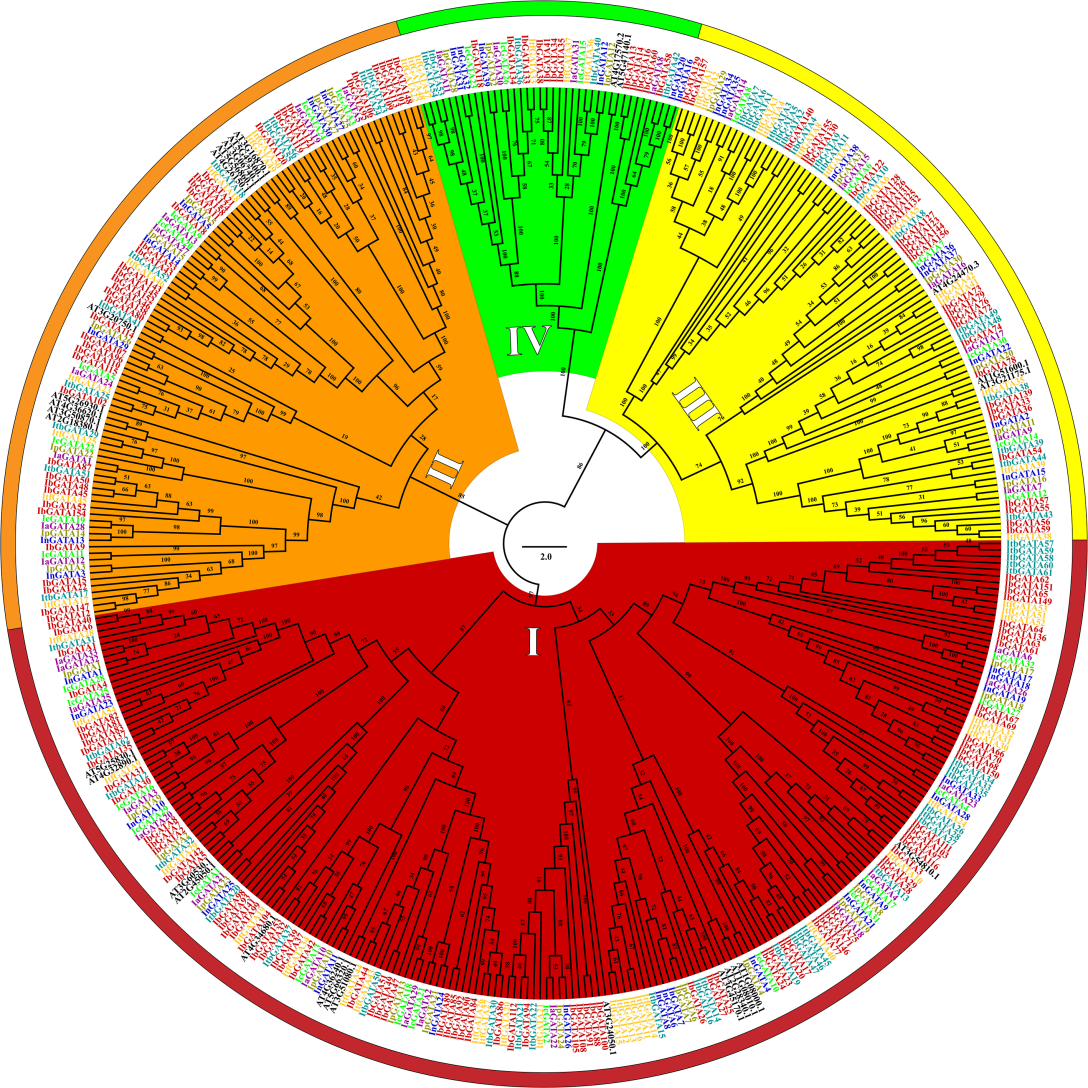
Phylogenetic tree of the *GATA* genes in sweetpotato, *I. trifida, I. triloba, I. nil, I. purpurea, I. cairica, I. aquatica*, and *Arabidopsis*. The *GATA* gene names of Sweetpotato, *I. trifida, I. triloba, I. nil, I. purpurea, I. cairica, I. aquatica*, and *Arabidopsis* were colored red, orange, teal, blue, brass, green, purple and black, respectively. Red, orange, yellow, and green represent the phylogenetic group I, II, III, and IV, respectively.

To elucidate the phylogenetic relationships of *GATA* genes in *Ipomoea* species, we constructed a phylogenetic tree using aligned protein sequences from 408 *Ipomoea GATA* genes (after excluding two problematic sweetpotato genes, *IbGATA10* and *IbGATA19*) and 29 *Arabidopsis thaliana* reference genes (**Figure 1**). The 437 analyzed genes clustered into four distinct groups (I-IV), with group I being predominant (208 genes, 47.60%), followed by group II (101, 23.11%), group III (88, 20.14%), and group IV (40, 9.15%). Species-specific distribution patterns revealed consistent grouping tendencies: sweetpotato (156 genes) showed 48.08% in group I, 25.64% in II, 18.59% in III, and 7.69% in IV; *I. trifida* (54 genes) distributed as 50.00%, 16.67%, 24.07%, and 9.26%; *I. triloba* (62 genes) as 46.77%, 17.74%, 27.42%, and 8.06%; *I. nil* (39 genes) as 46.15%, 17.95%, 20.51%, and 15.38%; while *I. purpurea*, *I. cairica* (each 32 genes), and *I. aquatica* (33 genes) exhibited similar distributions ranging 45.45-46.88% in group I, 24.24-25.00% in II, 18.18-18.75% in III, and 7.69-12.12% in IV.

### 2.3 Conserved motifs and structures of the *Ipomoea GATA* genes

**Figure 2:**
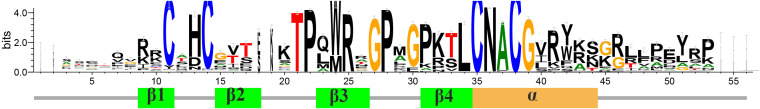
Logo plot and secondary structure annotation of the conserved GATA domain sequences.

To characterize GATA proteins in *Ipomoea* species, we analyzed their conserved domain sequences, revealing a type IV zinc finger motif (C-X_2_-C-X_18/20_-C-X_2_-C) similar to other plants (**Supplementary File 2**: **Supplementary Figure S1**). Group I, II, and IV proteins share a C-X_2_-C-X_18_-C-X_2_-C pattern, while class III uniquely possesses a C-X_2_-C-X_20_-C-X_2_-C variant (**Supplementary File 2**: **Supplementary Figure S1**). Beyond conserved cysteines, this domain exhibits multiple conserved residues potentially involved in *cis*-element recognition, along with group-specific amino acid variations that may reflect functional divergence (**Figure 2**). Structural analysis confirmed the conserved architecture of four β sheets and one α helix in *Ipomoea* GATA domains, consistent with *Arabidopsis* findings (**Figure 2**).

### 2.4 Structural and motif analysis of *GATA* genes in *Ipomoea* species

**Figure 3:**
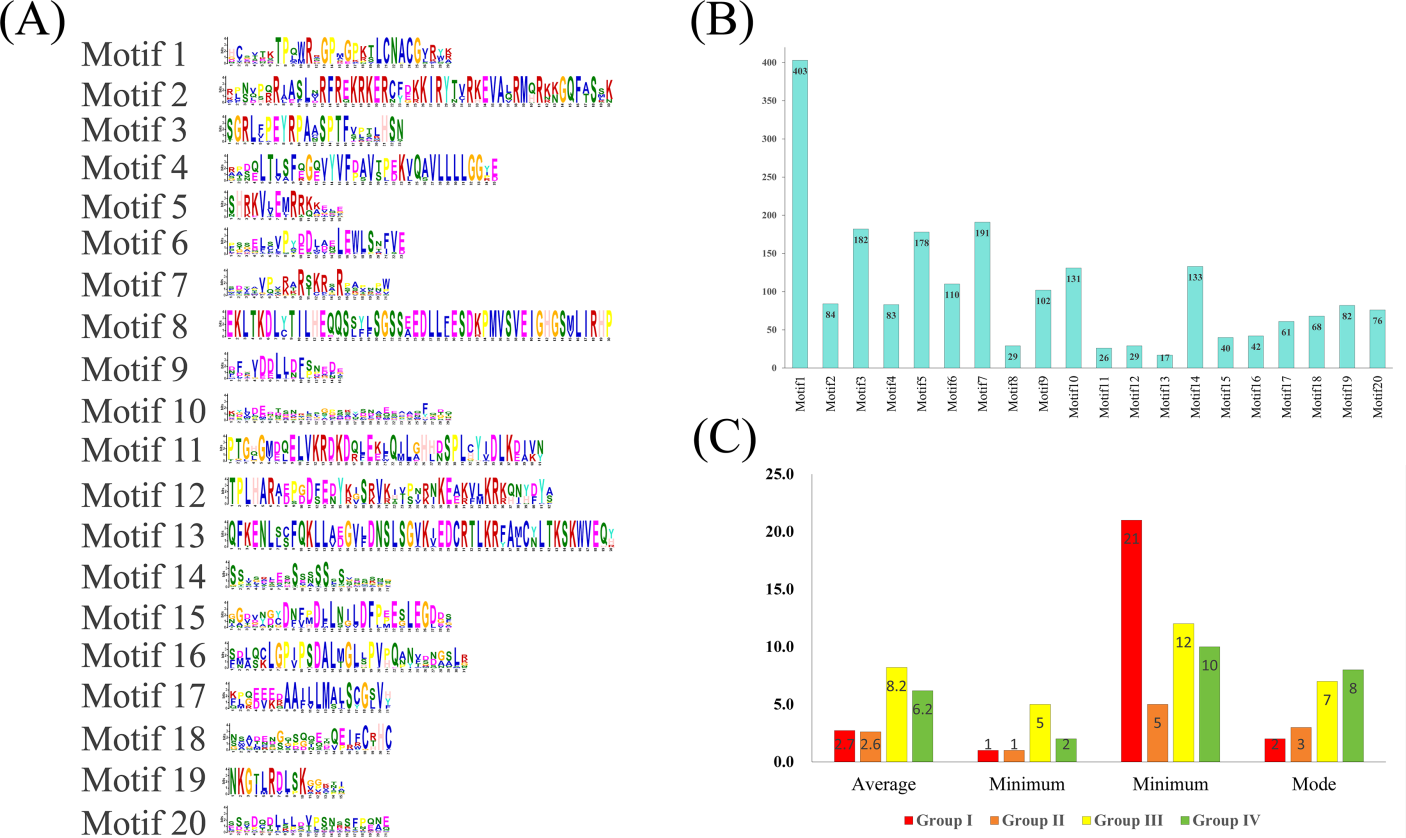
Structural characterization of *Ipomoea* GATA proteins. **(A)** Distribution of 20 conserved motifs (numbered 1-20) across protein sequences. **(B)** Frequency analysis of motif occurrence in protein sequences. **(C)** Exon architecture statistics (mean, range, and mode) across phylogenetic groups.

In *Ipomoea* GATA proteins, 20 distinct motifs were identified, with motif-1 (GATA domain) being the most prevalent (403 proteins, 98.77%) and conserved (**Figure 3**; **Supplementary File 3: Supplementary Figure S2**). Subsequent motifs showed decreasing frequencies: motif 7 (46.81%), motif 3 (44.61%), motif 5 (43.63%), motif 14 (32.60%), and motif 10 (32.11%). Phylogenetically related groups shared conserved motif patterns (**Supplementary File 3: Supplementary Figure S2**). Most *GATA* genes contained multiple exons (minimum one intron), with Groups I and II averaging 2.7 and 2.6 exons respectively. Group I predominantly contained 2-exon genes (43.81%), while Group II favored 3-exon configurations (45.41%). In contrast, Groups III and IV exhibited substantially higher exon counts (averaging 8.2 and 6.2 respectively), with 7-exon (42.35%) and 8-exon (50.00%) architectures being most common in each group (**Figure 3**; **Supplementary File 1: Supplementary Table S1**). Motif annotation revealed that most motifs lacked significant functional annotation, with three notable exceptions: motif 1 was identified as the GATA domain, motif 2 as the CCT motif, and motif 4 as the TIFY domain (**Supplementary File 4: Supplementary Table S2**).

### 2.5 Chromosomal location analysis of the *Ipomoea GATA* genes

**Figure 4:**
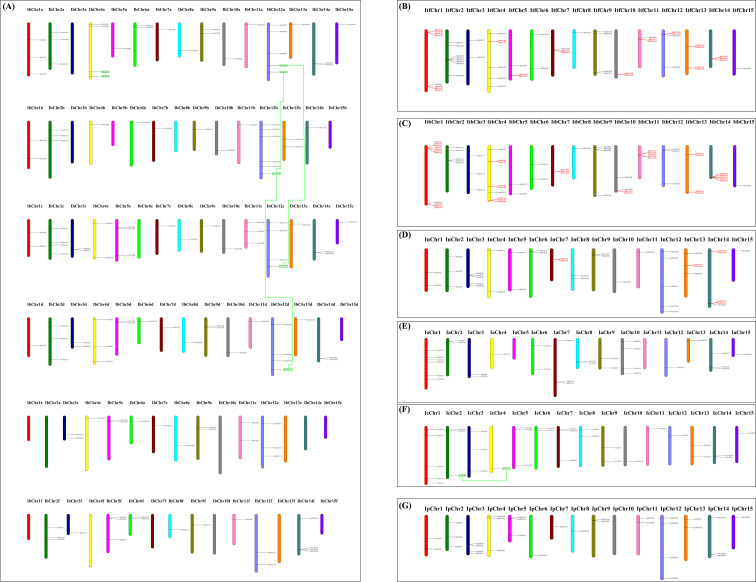
Distribution of *GATA* genes across the chromosomes of seven *Ipomoea* species. **(A)** Sweetpotato; **(B)***I*. *trifida*; **(C)***I*. *triloba*; **(D)***I*. *nil*.; **(E)***I*. *purpurea*; **(F)***I*. *cairica*; **(G)***I*. *aquatica*. The red color indicates the tandemly duplicated *GATA* genes; the green rectangular boxes connected by green lines indicates the segmentally duplicated *GATA* genes.

All *Ipomoea GATA* genes were successfully mapped across the chromosomes of seven *Ipomoea* species, with the exception of 24 *IbGATAs* and 3 *IaGATAs* located in unassembled scaffolds (**Figure 4**). The chromosomal distribution of these genes showed significant variation. In sweet potato (*Ipomoea batatas*), for example, chromosomes IbChr12b, IbChr12a, IbChr4a, IbChr2c, IbChr12c, IbChr12d, and IbChr14f contained 6, 5, 4, 4, 4, 4, and 4 *IbGATAs* respectively. In contrast, no *IbGATAs* were detected on multiple chromosomes including IbChr3a, IbChr3b, IbChr5b, IbChr8b, IbChr15b, IbChr6c, IbChr15d, and several chromosomes from the e and f series (IbChr1e to IbChr15f, excluding those already mentioned). This uneven distribution pattern was similarly observed in other *Ipomoea* species (**Figure 4**).

### 2.6 Duplication pattern analysis of the *Ipomoea GATA* genes

To investigate the evolutionary patterns of *Ipomoea GATA* genes, we analyzed gene duplication events using MCScanX software (**Figure 4**; **Supplementary File 5: Supplementary Table S3**). The analysis revealed tandem duplications in three species: 21 gene pairs in *I. triloba*, 15 in *I. trifida*, and 4 in *I. nil*, with no tandem duplications detected in other *Ipomoea* species. Segmentally duplicated *GATA* genes were found only in sweetpotato (4 pairs) and *I. cairica* (1 pair). Phylogenetic classification showed these duplicated genes distributed across four groups: 21 pairs (2 segmental, 19 tandem) in group I, 4 pairs (1 segmental, 3 tandem) in group II, 16 pairs (1 segmental, 15 tandem) in group III, and 4 pairs (1 segmental, 3 tandem) in group IV (**Figure 4**; **Supplementary File 5: Supplementary Table S3**).

### 2.7 Syntenic analysis of *GATA* genes in the genomes of the seven *Ipomoea* species

**Figure 5:**
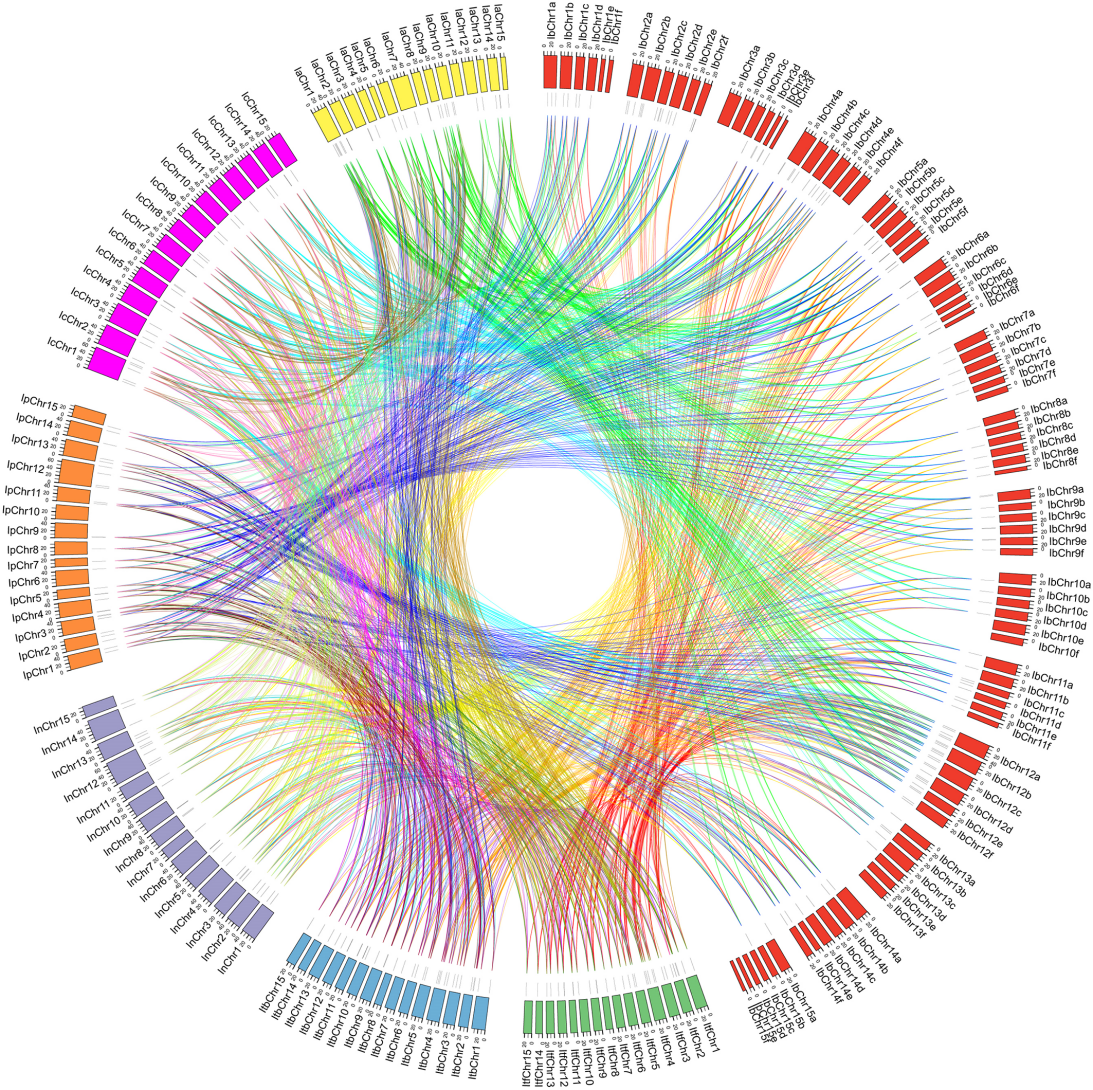
Syntenic analyses of *GATA* genes in the genomes of *Ipomoea* species. Chromosomal distribution in the seven *Ipomoea* species. The outer circle represents the haploid chromosomes of sweet potato (*I. batatas*) (red), *I. trifida* (green), *I. triloba* (cornflower blue), *I. nil* (medium purple), *I. purpurea* (orange), *I. cairica* (magenta) and *I. aquatica* (yellow), respectively. The second circle (black) represents the matches of *GATA* genes with the genome of the *Ipomoea* species. Colorful lines show the collinear *GATA* gene pairs in the whole genome of the *Ipomoea* species.

To determine the evolutionary mechanism of *Ipomoea GATA* genes, comparative synteny maps of the seven *Ipomoea* species were constructed (**Figure 5**). A total of 321 *Ipomoea GATA* genes (124 *IbGATAs*, 35 *ItfGATAs*, 37 *ItbGATAs*, 32 *InGATAs*, 31 *IpGATAs*, 32 *IcGATAs*, and 30 *IaGATAs*) that formed 2104 ortholog pairs were detected in the seven *Ipomoea* species (**Figure 5**; **Supplementary File 6: Supplementary Table S4**). Of these ortholog pairs, sweetpotato and *I. cairica* harbored the most ortholog *GATA* gene pairs (226 pairs), followed by sweetpotato and *I. aquatica* (214 pairs), sweetpotato and *I. purpurea* (196 pairs), sweetpotato and *I. trifida* (190 pairs), sweetpotato and *I. triloba* (185 pairs), sweetpotato and *I. nil* (169 pairs), *I. trifida* and *I. cairica* (128 pairs), and the others (50–66 pairs). The ortholog *GATA* genes were distributed in all of the subfamilies (group I-IV) (**Supplementary File 6: Supplementary Table S4**). In most cases (1759 of 2104, 83.60%), the two of ortholog *GATA* genes were from the same subfamily (**Supplementary File 6: Supplementary Table S4**).

**Figure 6:**
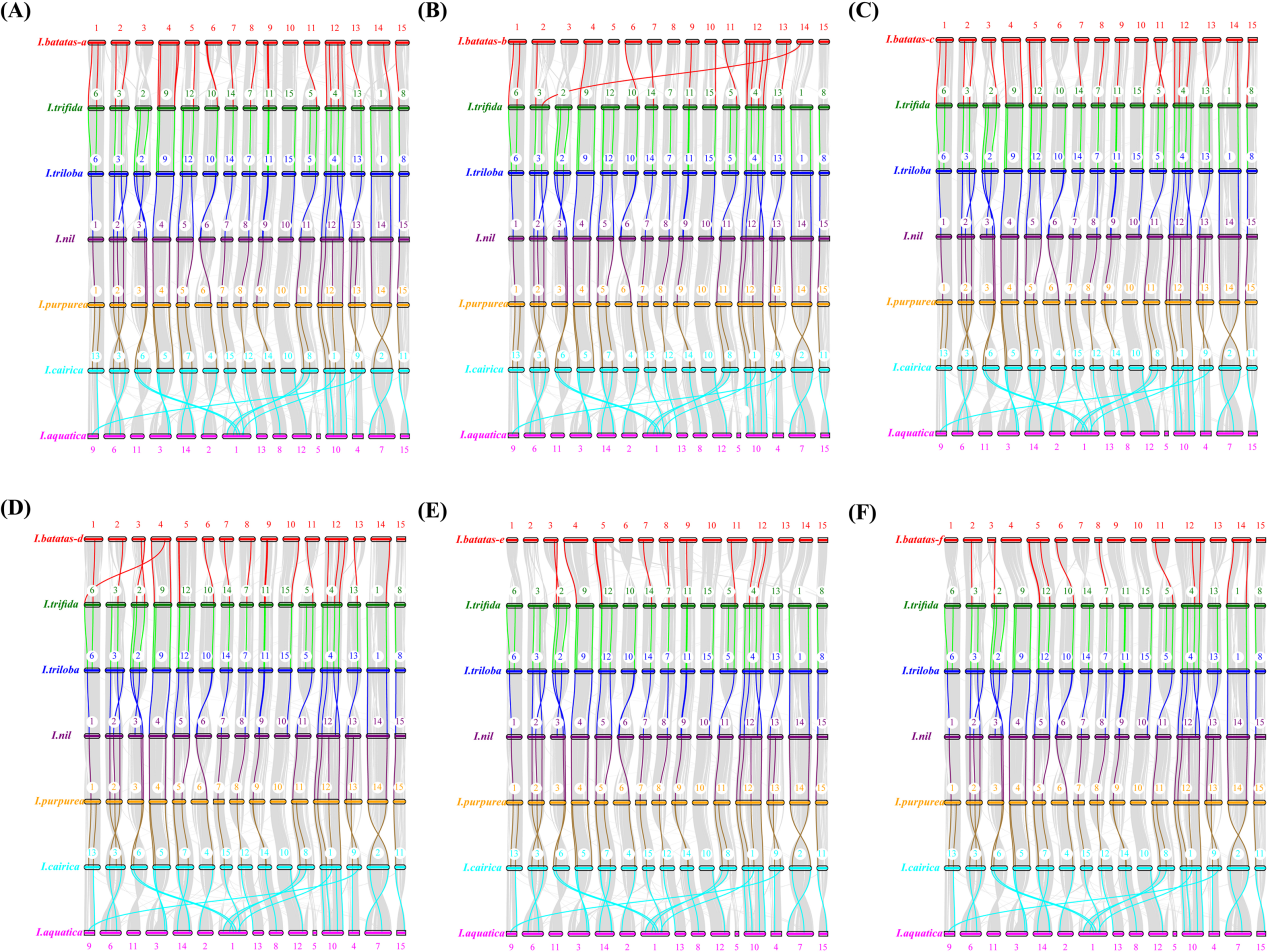
Schematic representation of syntenic genes among sweet potato (*I. batatas*), *I. trifida*, *I. triloba*, *I. nil*, *I. purpurea*, *I. cairica* and *I. aquatica*. **(A–F)** Schematic representation of syntenic genes among **(A–F)** sub-genome of sweetpotato, *I. trifida*, *I. triloba*, *I. nil*, *I. purpurea*, *I. cairica* and *I. aquatica*, respectively. The chromosomes of the seven *Ipomoea* species were reordered through collinearity for observation. The chromosomes of sweetpotato, *I. trifida*, *I. triloba*, and *I. nil* were colored with red, green, blue, purple, orange, cyan, and pink, respectively. Gray lines connect matched gene pairs, with *GATA* gene pairs highlighted in red, green, blue, purple, orange, and cyan, respectively.

A total of 199 *GATA* genes (79 from sweet potato, 20 from *I. trifida*, 20 from *I. triloba*, 20 from *I. nil*, 20 from *I. purpurea*, 20 from *I. cairica*, and 20 from *I. aquatica*) were identified as orthologous gene pairs among *Ipomoea* species (**Figure 6**; **Supplementary File 7: Supplementary Table S5**). Among these, 84 genes (42.21%) belonged to phylogenetic group I, 59 (29.65%) to group II, 48 (24.12%) to group III, and 8 (4.02%) to group IV (**Supplementary File 7: Supplementary Table S5**). Of the 79 sweet potato *GATA* genes, 17 originated from sub-genome A, 11 from B, 16 from C, 14 from D, 10 from E, and 11 from F (**Figure 6**; **Supplementary File 7: Supplementary Table S5**).

### 2.8 Ka/Ks analysis of duplicated and syntenic *Ipomoea GATA* genes

To detect whether duplicate and syntenic *GATA* genes were under positive selection, Ka/Ks analysis was performed (**Supplementary File 8: Supplementary Table S6**). A total of 2149 gene pairs (5 segmental duplicated pairs, 40 segmental duplicated pairs, and 2104 collinear gene pairs) were analyzed, with Ka/Ks ratios successfully calculated for 1804 (83.94%) of them (**Supplementary File 8: Supplementary Table S6**). All duplicated and syntenic *GATA* genes, except for one tandem duplicated gene pair from *I. trifida* (*ItfGATA20*-*ItfGATA21*, Ka/Ks = 1.01), showed Ka/Ks ratios below one, suggesting that most had undergone purifying selection.

### 2.9 Stress-related regulatory elements analysis in promoter regions of the *Ipomoea GATA* genes

The 1,500 bp upstream regulatory regions of all *Ipomoea GATA* genes were used to explore stress-related regulatory elements. Various elements were detected. In this present investigation, ABRE, ARE, CGTCA-motif, GC-motif, LTR, MBS, TCA-element, TC-rich repeats, TGACG-motif were calculated (**Supplementary File 9: Supplementary Figure S3**). A total of 3552 elements in 398 *Ipomoea GATA* genes’ promoter regions were detected (**Supplementary File 10: Supplementary Table S7**). Of them, the largest one was ABRE (#819), followed by ARE (#758), TGACG-motif (#461), CGTCA-motif (#461), MBS (#341), TCA-element (#250), LTR (#216), GC-motif (#111), and TC-rich repeats (#105). The average investigated *cis*-element number of the *Ipomoea GATA* is 8.87 (**Table 1**). When compared the average investigated *cis*-element number of the phylogenetic group, group II was the largest (10.00), followed by group I (8.85), III (8.52), and IV (7.08). The average ABRE, CGTCA-motif, GC-motif, MBS, and TGACG-motif in the group II *Ipomoea GATA* genes’ promoter region were relatively larger than that in other groups (**Table 1**).

**Table 1:** The average *cis*-elements number in each phylogenetic group genes.

Phylogenetic group	I	II	III	IV	Overall
ABRE	2.21	2.55	1.78	0.86	2.07
ARE	1.62	2.02	2.35	2.05	1.90
CGTCA-motif	1.15	1.34	0.98	1.16	1.16
GC-motif	0.31	0.30	0.27	0.14	0.28
LTR	0.58	0.44	0.60	0.49	0.55
MBS	0.86	1.06	0.78	0.57	0.86
TCA-element	0.69	0.67	0.63	0.27	0.63
TC-rich repeats	0.28	0.28	0.16	0.38	0.27
TGACG-motif	1.15	1.34	0.98	1.16	1.16
Overall	8.85	10.00	8.52	7.08	8.87

### 2.10 Expression patterns of the *GATA* genes in the sweetpotato

**Figure 7:**
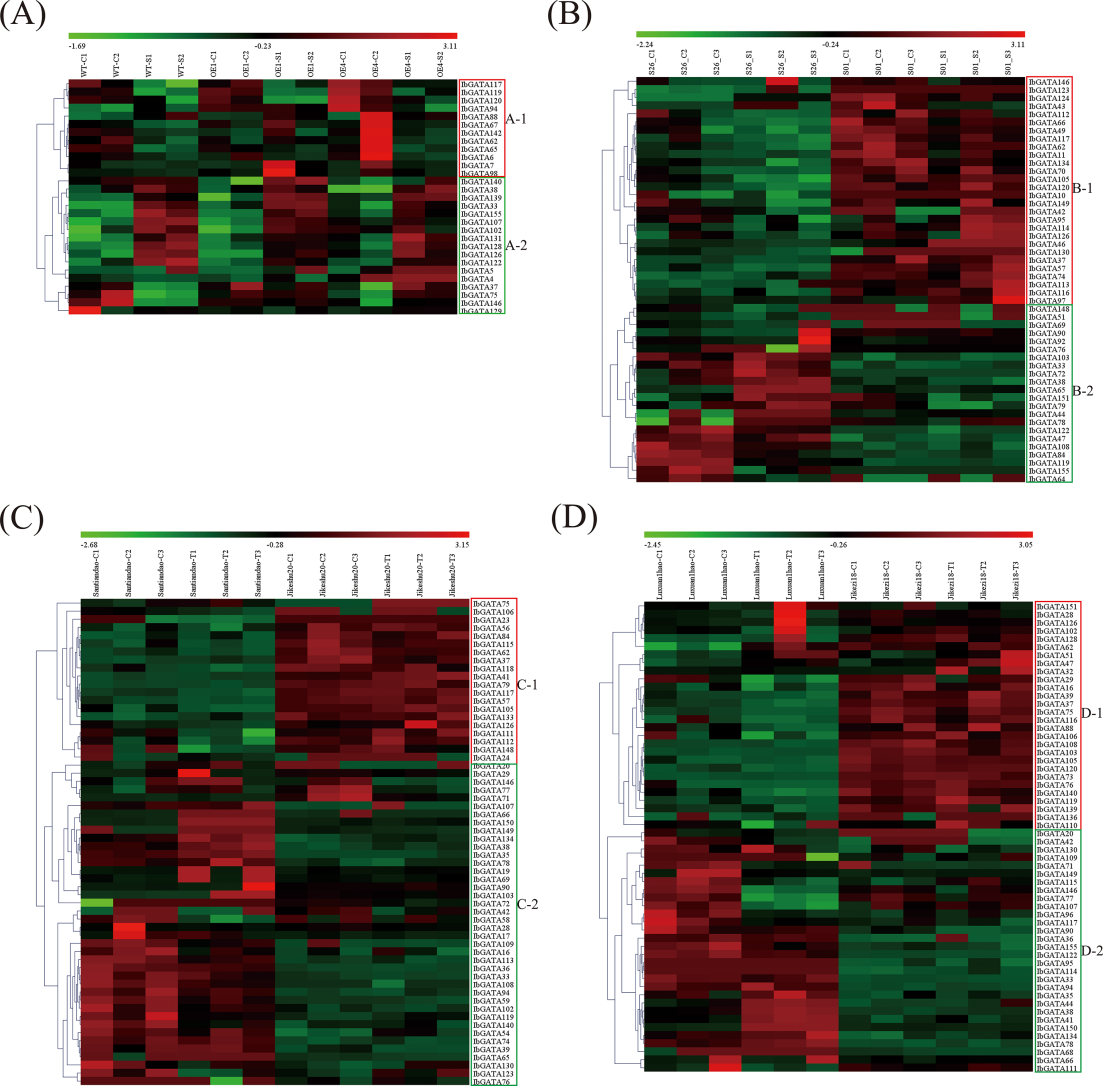
Heatmap of the expression profiles of sweetpotato differentially expressed genes (DEGs) in response to biotic and abiotic stresses. **(A)** DEGs in “WT”, “OE1” and “OE4” under control and salt treatment. **(B)** DEGs in “S26” and “S01” under control and drought treatment. **(C)** DEGs in “Santiandao” and “Jikeshu20” under control and *Ceratocystis fimbriata* infection treatment. **(D)** DEGs in “Luxuan1hao” and “Jikezi18” under control and *Ditylenchus destructor* infection treatment. C, control; T, treatment.

To explore *GATA* genes related to stress response, four transcriptome datasets covering abiotic stresses (salt and drought treatments) and biotic stresses (*Ceratocystis fimbriata* and *Ditylenchus destructor* infections) were analyzed (**Figure 7**). In the salt stress analysis, 29 *GATA* differentially expressed genes (DEGs) were identified and classified into two subclasses (A-1 and A-2) based on expression patterns (**Figure 7A**). Subclass A-1 (12 *IbGATAs*) showed predominant upregulation in controls but downregulation under salt stress, whereas subclass A-2 (17 *IbGATAs*) exhibited the opposite trend (**Figure 7A**). The drought stress analysis revealed 50 *GATA* DEGs, divided into subclasses B-1 (28 *IbGATAs*) and B-2 (22 *IbGATAs*). Subclass B-1 genes were downregulated in both control and stressed conditions of drought-sensitive genotype S26, but upregulated in drought-resistant genotype S01. Conversely, subclass B-2 genes displayed inverse expression patterns (**Figure 7B**). For *Ceratocystis fimbriata* infection, 60 *GATA* DEGs were grouped into subclasses C-1 (20 *IbGATAs*) and C-2 (40 *IbGATAs*). Subclass C-1 was downregulated in susceptible genotype Santiandao but upregulated in resistant Jikeshu20, with subclass C-2 showing reciprocal regulation (**Figure 7C**). The *Ditylenchus destructor* infection analysis detected 58 *GATA* DEGs, categorized into subclasses D-1 (28 *IbGATAs*) and D-2 (30 *IbGATAs*). Similar differential expression patterns were observed between susceptible Luxuan1hao and resistant Jikezi18 genotypes (**Figure 7D**). Cross-analysis identified 8 consistently detected DEGs (*IbGATA117/119/126/146/33/37/38/62*) across all datasets.

### 2.11 Expression analysis of sweetpotato *GATA* genes by quantitative reverse-transcription polymerase chain reaction

**Figure 8:**
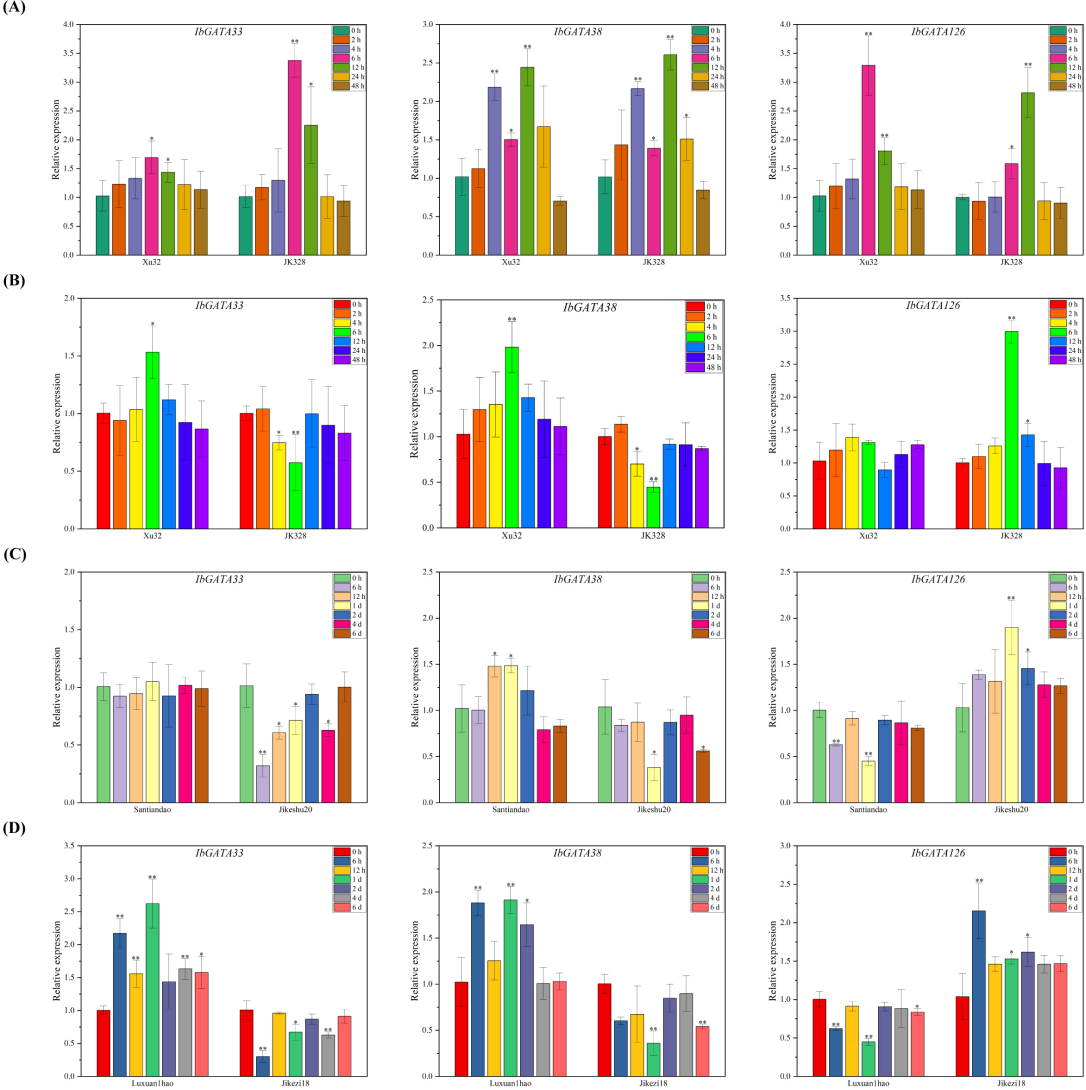
Expression analysis of *IbGATA33, IbGATA38*, and *IbGATA126* in sweetpotato cultivars or lines. **(A)** Relative expression levels in leaves after different times of salt (86 mM NaCl) treatments. **(B)** Relative expression levels in leaves after different times of drought (30% PEG 6000) treatments. **(C)** Relative expression levels after different times of *Ceratocystis fimbriata* infection. **(D)** Relative expression levels in storage roots after *Ditylenchus destructor* infection. Denoted the significance of expression levels compared with control were as ∗<0.05, ∗∗<0.01. h, hours; d, day(s).

Based on transcriptome results, *IbGATA33*, *IbGATA38*, and *IbGATA126* were selected for further analysis using qRT-PCR (**Figure 8**). Compared with the control condition (0 h), the transcripts of *IbGATA33*, *IbGATA38*, and *IbGATA126* in Xushu32 were all upregulated after salt treatments, reaching peaks at 6 h (1.69-fold), 12 h (2.44-fold), and 6 h (3.29-fold), respectively; the transcripts of *IbGATA126*, *IbGATA33*, and *IbGATA38* in JK328 were all upregulated after salt treatments, reaching peaks at 6 h (3.38-fold), 12 h (2.61-fold), and 12 h (2.81-fold), respectively (**Figure 8A**). Compared with the control condition (0 h), the transcripts of *IbGATA33* and *IbGATA38* in Xushu32 were upregulated after drought treatments, peaking at 6 h (1.53-fold) and 6 h (1.98-fold), respectively, while no significant change was observed in *IbGATA126* transcripts; the transcripts of *IbGATA33* and *IbGATA38* in JK328 were downregulated after drought treatments, reaching lowest levels at 6 h (0.57-fold) and 6 h (0.44-fold), respectively, while *IbGATA126* transcripts were upregulated, peaking at 6 h (2.99-fold) (**Figure 8B**). Compared with the control condition (0 h), *IbGATA38* transcripts in Santiandao were upregulated after *Ceratocystis fimbriata* infection, peaking at 1 d (1.49-fold), while *IbGATA126* transcripts were downregulated, reaching the lowest level at 1 d (0.44-fold), with no significant change in *IbGATA33* transcripts. In Jikeshu20, *IbGATA33* and *IbGATA38* transcripts were downregulated after *Ceratocystis fimbriata* infection, reaching lowest levels at 6 h (0.32-fold) and 1 d (0.38-fold), respectively, while *IbGATA126* transcripts were upregulated, peaking at 1 d (1.90-fold) (**Figure 8C**). Compared with the control condition (0 h), *IbGATA33* and *IbGATA38* transcripts in Luxuan1hao were upregulated after *Ditylenchus destructor* infection, peaking at 1 d (2.62-fold and 1.91-fold, respectively), while *IbGATA126* transcripts were downregulated, reaching the lowest level at 1 d (0.45-fold). In Jikezi18, *IbGATA33* and *IbGATA38* transcripts were downregulated after *Ditylenchus destructor* infection, reaching lowest levels at 6 h (0.30-fold) and 1 d (0.36-fold), respectively, while *IbGATA126* transcripts were upregulated, peaking at 6 h (2.15-fold) (**Figure 8D**).

## 3 Discussion

Plant GATA transcription factors are key regulatory proteins governing growth, development, and environmental adaptation (Schwechheimer et al., 2022). Consequently, this gene family has garnered increasing research interest and undergone comprehensive characterization across diverse plant species, including *Arabidopsis* (Reyes et al., 2004), rice (*Oryza sativa*) (Reyes et al., 2004), wheat (*Triticum aestivum*) (Feng et al., 2022), potato (*Solanum tuberosum*) (Zhang et al., 2024), poplar (*Populus* spp.) (Zhao et al., 2023), melon (*Cucumis melo*) (Zheng et al., 2024b), onion (*Allium cepa*) (Bose et al., 2025), Solanaceae species (Zhang et al., 2023), Orchidaceae species (Zheng et al., 2024c), and *Populus* species (Kim et al., 2021b). The genus *Ipomoea* comprises 600–700 species, many of which have significant medicinal or ornamental value (Nimmakayala et al., 2011). However, the *GATA* gene family remains poorly characterized in *Ipomoea* species.

In the present study, we identified a total of 410 *GATA* genes across seven *Ipomoea* species. Analysis revealed variations in *GATA* gene family members among the studied species: sweet potato (*I. batatas*) contained 158 genes (0.09%), *I. trifida* 54 (0.12%), *I. triloba* 62 (0.13%), *I. nil* 39 (0.09%), *I. purpurea* 32 (0.10%), *I. cairica* 32 (0.08%), and *I. aquatica* 33 (0.06%). The corresponding genome sizes were 2,907.4 Mb (*I. batatas*), 373.4 Mb (*I. trifida*), 443.3 Mb (*I. triloba*), 750.0 Mb (*I. nil*), 602.0 Mb (*I. purpurea*), 733.0 Mb (*I. cairica*), and 511.5 Mb (*I. aquatica*). These findings suggest that *GATA* gene quantity is independent of genome size, a phenomenon previously observed in closely related species including Solanaceae (Zhang et al., 2023), Orchidaceae (Zheng et al., 2024c), and *Populus* (Kim et al., 2021b).

The phylogenetic analysis of *GATA* genes in seven *Ipomoea* species and *Arabidopsis* revealed four independent groups (I to IV) (**Figure 1**), consistent with findings in other plant species (Manzoor et al., 2021; Shi et al., 2022; Zhang et al., 2023). Compared to other plant species, the proportion of *GATA* genes in each phylogenetic group was distinct when using *Arabidopsis GATA* genes as a reference. For instance, in Rosaceae species, Group IV was the largest and Group I was the smallest (Manzoor et al., 2021); in longan and apple, Group II was the largest and Group I was the smallest (Zheng et al., 2024a); in *Populus* species and wheat, the distribution was similar to *Ipomoea* species, with Group I being the largest and Group IV the smallest (Kim et al., 2021b; Feng et al., 2022; Zhao et al., 2023). These results revealed that the ancestral *GATA* gene phylogenetic groups have undergone different expansion patterns across plant species (Lespinet et al., 2002).

The conserved GATA domain in *Ipomoea* comprises four β-sheets and one α-helix, featuring a type IV zinc finger motif (C-X_2_-C-X_18/20_-C-X_2_-C) (**Supplementary File 2: Supplementary Figure S1**, **Figure 2**). Group I, II, and IV proteins share a C-X_2_-C-X_18_-C-X_2_-C pattern, while class III uniquely possesses a C-X_2_-C-X_20_-C-X_2_-C variant (**Supplementary File 2: Supplementary Figure S1**). These findings align with conserved structural features observed in other plant species, including *Arabidopsis* (Reyes et al., 2004; Bi et al., 2005; Kim et al., 2021a), poplar (Zhao et al., 2023), wheat (Feng et al., 2022), and rice (Gupta et al., 2017). Through motif analysis and annotation of *Ipomoea* GATA proteins, 20 conserved motifs were identified (**Figure 3**; **Supplementary File 4: Supplementary Table S2**). Among these, only three motifs were annotated as GATA, CCT, and TIFY, respectively. Notably, motif 1 corresponds to the GATA domain, while the remaining motifs exhibit class-specific distribution patterns, suggesting potential functional diversification within this protein family. Consistent with findings in other plant species, CCT motifs are exclusively present in Group III, whereas TIFY motifs are restricted to Groups III and IV (**Figure 3**; **Supplementary File 4: Supplementary Table S2**). While the precise function of the CCT motif remains unclear, proteins containing this motif have been implicated in photoperiod sensing and circadian rhythm integration (Schwechheimer et al., 2022). In contrast, the TIFY motif is well-documented to participate in biological clock regulation and hormone signaling pathways (Peng et al., 2021).

Gene duplication events, including segmental and tandem duplications, play essential roles in gene family expansion and distribution in plants (Cannon et al., 2004; Kong et al., 2007; Jiang et al., 2013). Segmental duplications typically occur through polyploidy followed by chromosomal rearrangements, while tandem duplications arise within the same or neighboring intergenic regions (Jiang et al., 2013). In this study, the distribution of *Ipomoea GATA* genes was found to be uneven across chromosomes (**Figure 4**). Tandem duplications were detected only in *I. triloba*, *I. trifida*, and *I. nil*, while segmentally duplicated *GATA* genes were observed in sweetpotato and *I. cairica* (**Figure 4**; **Supplementary File 5: Supplementary Table S3**). These results suggest that *Ipomoea GATA* genes may have experienced distinct duplication mechanisms compared to other plant lineages (Kong et al., 2007).

This study identified 199 *GATA* orthologous genes across seven *Ipomoea* species, including 79 from sweet potato and 20 each from *I. trifida*, *I. triloba*, *I. nil*, *I. purpurea*, *I. cairica*, and *I. aquatica*. Synteny analysis of *GATA* genes in the seven *Ipomoea* species revealed strong collinearity despite chromosomal rearrangements and gene duplication events following divergence from their common ancestor (Yan et al., 2022). To elucidate the evolutionary dynamics of duplicated and syntenic *GATA* gene pairs, we conducted Ka/Ks analysis. The results demonstrated that nearly all *GATA* gene pairs exhibited a Ka/Ks ratio below 1, indicating predominant purifying (negative) selection during genome duplication and speciation events (Gaut and Doebley, 1997).

Regulatory elements are specific DNA sequences within the same DNA molecule that possess transcriptional regulation functions. Analyzing these elements can enhance our fundamental understanding of gene regulation (Baxter et al., 2012; Hernandez-Garcia and Finer, 2014). As anticipated, the promoters of the *Ipomoea GATA* genes contained numerous *cis*-regulatory elements involved in biotic and abiotic stress responses. These included ABRE, ARE, CGTCA-motif, GC-motif, LTR, MBS, TCA-element, TC-rich repeats, and TGACG-motif (**Supplementary File 9: Supplementary Figure S3, Supplementray File 10:****Supplementary Table S7**). The abundance of these stress-related regulatory elements likely explains why a large proportion of *GATA* genes showed stress-responsive expression patterns in our analysis.

Research has reported that *GATA* genes participate in both plant developmental processes (Wang et al., 2009; Luo et al., 2010; Lu et al., 2017; Zhang et al., 2018, 2020) and stress response mechanisms (Zhao et al., 2021b; Zhu et al., 2022; Wei et al., 2023). In this study, we examined *GATA* gene expression patterns through analysis of RNA-seq data. Differential expression profiles were observed, with *Ipomoea GATA* genes showing distinct stress-responsive expression patterns (**Figure 7**). For stress response analysis, we selected four RNA-seq datasets comprising two abiotic (salt, drought) and two biotic (*Ceratocystis fimbriata*, *Ditylenchus destructor*) stress conditions. This analysis identified 29, 50, 60, and 58 differentially expressed *GATA* genes (DEGs) respectively (**Figure 7**). Subsequent qRT-PCR validation of three selected genes (*IbGATA33*, *IbGATA38*, *IbGATA126*) confirmed the RNA-seq expression patterns (**Figure 8**). The qRT-PCR validation revealed differential expression patterns of *IbGATA33/38/126* genes. Under salt stress, all three genes were upregulated in both Xushu32 and JK328 cultivars, peaking at 6 h or 12 h (1.69- to 3.38-fold increase). During drought treatment, *IbGATA33/38* were upregulated in Xushu32 but downregulated in JK328, whereas *IbGATA126* exhibited opposite expression trends between the two cultivars. In pathogen responses: *C. fimbriata* infection induced upregulation of *IbGATA38* (1.49-fold) and downregulation of *IbGATA126* (0.44-fold) in Santiandao; *D. destructor* infection caused upregulation of *IbGATA33/38* (1.91- to 2.62-fold) and downregulation of *IbGATA126* (0.45-fold) in Luxuan1hao, while Jikezi18 displayed divergent trends. Based on these results, it is possible to predict that *IbGATA33*, *IbGATA38*, and *IbGATA126* play important roles in abiotic and biotic stress responses, and their functions should be investigated in the near future.

## 4 Conclusions

We analyzed *GATA* genes in seven *Ipomoea* species (*I. batatas*:158, *I. trifida*:54, *I. triloba*:62, *I. nil*:39, *I. purpurea*:32, *I. cairica*:32, *I. aquatica*:33), classifying them into four clades (I-IV). Conserved motifs, gene structures, and chromosomal distributions were characterized, revealing tandem and segmental duplications drove family expansion. Among 199 orthologs, syntenic pairs showed Ka/Ks<1, indicating purifying selection. Stress treatments identified 29–60 differentially expressed *GATA* genes (salt/drought/pathogens). qRT-PCR validated three DEGs (*IbGATA33*, *IbGATA38*, *IbGATA126*), confirming transcriptome data. These results provide a comprehensive genomic analysis of the GATA transcription factor family across seven *Ipomoea* species, offering valuable insights into gene characteristics, phylogenetic relationships, chromosomal locations, duplication events, *cis*-regulatory elements, expression patterns, and stress responses. This analysis may facilitate the elucidation of evolutionary relationships, molecular mechanisms, and functional roles of *GATA* genes in *Ipomoea* species.

## 5 Methods

### 5.1 Data resources

Genomic data for seven *Ipomoea* species were obtained from public databases: sweetpotato genome (version 1) from Plant GARDEN (Yoon et al., 2022), *I. trifida* (v3) and *I. triloba* (v3) from GenBank BioProject PRJNA428214 and PRJNA428241 respectively (Wu et al., 2018), *I. nil* (v1.2) from GenBank BioProject BDFN01000001-BDFN01003416 (Hoshino et al., 2016), *I. purpurea* (v1) from CoGe platform (Zhao et al., 2021a), *I. cairica* (v1) from AGIS database (Jiang et al., 2022), and *I. aquatica* (v1) from BIGD (PRJCA002216) (Hao et al., 2021). *Arabidopsis* GATA protein sequences were acquired from TAIR (Reyes et al., 2004).

### 5.2 Identification of *GATA* genes in seven *Ipomoea* species

The identification of GATA domains was conducted through a dual-algorithm strategy. Initial screening was performed using HMMER 3.1b2 with default parameters to detect the conserved GATA domain (Pfam: PF00320) in all protein sequences. In parallel, BLASTP 2.2.28+ searches were executed using an extended GATA domain sequence as query (E-value cutoff: 1×10^−10^). Candidate sequences from both HMMsearch and BLASTP analyses were merged, and redundancy was eliminated through sequence identity clustering. Final validation involved HMMscan verification of putative GATA proteins against the Pfam-A database with a strict E-value threshold of 0.0001.

### 5.3 Molecular weight, isoelectric point and subcellular localization analysis of *Ipomoea* GATA proteins

The ExPASy proteomics server (http://www.expasy.ch/tools/pi_tool.html) was utilized to calculate key physicochemical parameters of GATA proteins, specifically molecular weight (MW) and isoelectric point (pI) (Artimo et al., 2012). For subcellular localization prediction of *Ipomoea* proteins, we employed WoLF PSORT (https://wolfpsort.hgc.jp/), a dedicated bioinformatics platform for protein localization analysis (Horton et al., 2007).

### 5.4 Sequence alignment and phylogenetic analysis of GATA proteins

To reconstruct the phylogenetic relationships among identified GATA proteins, initial multiple sequence alignment of complete protein sequences was executed using Clustal Omega (v1.2.4) (Sievers et al., 2011; Sievers and Higgins, 2018). The alignment output served as input for maximum likelihood analysis performed with IQ-TREE (v2.1.3) (Minh et al., 2020), incorporating model selection via ModelFinder (v2.0) (Kalyaanamoorthy et al., 2017) that determined the VT+F+R4 model as most appropriate. Tree topology robustness was evaluated through SH-aLRT and UFBoot2 analyses (1,000 replicates). Final tree visualization and annotation were accomplished using FigTree (v1.4.3) to optimize clarity.

### 5.5 Identification of conserved motifs of the *GATA* genes

To examine the structural motif diversity among the identified *GATA* genes, their protein sequences underwent thorough motif analysis via the web-based platform MEME SUITE (v5.5.3), available at https://meme-suite.org/meme/ (Bailey et al., 2009). The analysis was designed to detect a maximum of 20 unique motifs, with site distribution set to “any” (permitting motif occurrence at any sequence position). Default values were retained for all other parameters to maintain methodological consistency and alignment with conventional approaches. *Ipomoea* GATA protein conserved domains were aligned and graphically represented using Clustal Omega (v1.2.4) (Sievers et al., 2011; Sievers and Higgins, 2018). Further sequence conservation analysis and GATA domain secondary structure visualization were performed through WebLogo (v3.7.9) (Crooks et al., 2004).

### 5.6 Protein motif compositions and gene structures of *Ipomoea GATA* genes

Based on the motif analysis data obtained from MEME SUITE (v5.5.3) (with the minimum width of 6, maximum width of 20, the maximum number of motifs designed to identify 20 motifs and iterative cycles set to default), phylogenetic relationships, and genome annotation files (gff3), the identified *Ipomoea GATA* genes were analyzed using TBtools-II (v2.131) to determine their protein motif distributions and gene structures, with subsequent graphical representation (Chen et al., 2023).

### 5.7 Chromosome distribution and duplication pattern analysis of the *GATA* genes

Chromosomal localization of *GATA* genes across all seven *Ipomoea* species was executed using MapChart (v2.30) (Voorrips, 2002). To detect putative gene duplication events, genome-wide collinearity assessments were carried out with MCScanX (Wang et al., 2012). This process included intra-species protein sequence comparisons via BLASTP (v2.2.28+) under a strict E-value threshold (1e-10). Synteny relationships were graphically rendered using CIRCOS (v0.66) to produce detailed genomic maps (Krzywinski et al., 2009).

### 5.8 Syntenic analysis *GATA* genes in the seven *Ipomoea* genomes

We conducted comparative synteny analysis of the seven *Ipomoea* species with MCScan (Python version) under default parameters (Tang et al., 2024). High-confidence 1:1 syntenic blocks (gene pairs) were identified through gene model alignments generated by LAST (v1257) and stringent filtering. The JCVI package (Tang et al., 2015) was employed to visualize syntenic relationships as dot plots.

### 5.9 Ka/Ks analysis of duplicated and syntenic *GATA* genes

The evolutionary selection pressures on GATA transcription factors were evaluated by determining the nonsynonymous (Ka) to synonymous (Ks) substitution rate ratio (ω = Ka/Ks) for duplicated and syntenic gene pairs in seven *Ipomoea* species, employing TBtools (v1.108) (Chen et al., 2020).

### 5.10 Promoter analysis of *GATA* genes in the seven *Ipomoea* species

To detect potential *cis*-elements in the *Ipomoea GATA* genes, their 1,500-bp promoter sequences were analyzed using PLANTCARE (http://bioinformatics.psb.ugent.be/webtools/plantcare/html/, accessed 18 March 2023) (Lescot et al., 2002).

### 5.11 Expression profile of sweetpotato *GATA* genes

To analyze the expression patterns of sweet potato *GATA* genes, four transcriptome datasets—covering both abiotic and biotic stresses—were utilized. Two abiotic stress datasets (salt: PRJNA811431; drought: PRJNA999504) were sourced from NCBI, while two unpublished in-house datasets investigated resistance to *C. fimbriata* and *D. destructor* across four cultivars/lines: the susceptible “Santiandao” and resistant “Jikeshu20” for *C. fimbriata*, and the susceptible “Luxuan1hao” and resistant “Jikezi18” for *D. destructor*. Differentially expressed genes (DEGs) were defined by |log2FC| > 1 and FDR ≤ 5%, with mean log2FC values computed for each. Expression distributions were visualized via an FPKM-based heat map generated in MeV software (Howe et al., 2011).

### 5.12 RNA isolation and qRT-PCR analysis

Two groups of sweet potato cultivars underwent distinct stress treatments. For biotic stress assessment, the susceptible cultivar Santiandao and resistant line Jikeshu20 were infected with *C. fimbriata* (Muramoto et al., 2012), whereas the susceptible Luxuan1hao and resistant Jikezi18 were inoculated with *D. destructor* (Gao et al., 2011). Samples were harvested at seven post-inoculation intervals (0, 6, 12 hours; 1, 2, 4, 6 days), with uninoculated roots as controls. For abiotic stress, pre-cultured cuttings (25 cm, from 6-week-old field plants) of susceptible Xu32 and resistant JK328 were treated in Hoagland solution for three days before exposure to salt (86 mM NaCl vs 0 mM), or drought (30% PEG6000 vs 0%) (Hoagland and Arnon, 1950). Abiotic samples were collected at seven time points (0–48 hours). Total RNA was isolated via RNAprep Pure Plant Kit (Tiangen Biotech) and reverse-transcribed using Quantscript RT Kit (Tiangen Biotech). The stably expressed β-actin gene (Genbank AY905538) normalized DEG expression. All experiments included triplicate biological replicates per time point, with gene expression analyzed by the 2^–ΔΔCt^ method (Schmittgen and Livak, 2008), and performed statistical analysis with one-way ANOVA. qRT-PCR followed published protocols, employing Primer-BLAST-designed primers (**Supplementary File 11: Supplementary Table S8**) (Ye et al., 2012; Zhai et al., 2016).

## Data Availability

The datasets presented in this study can be found in online repositories. The names of the repository/repositories and accession number(s) can be found in the article/**Supplementary Material**.

## Supplementary material

Supplementary Figure 1 Multiple sequence alignment of conserved GATA domains in *Ipomoea* species. Identical or similar amino acid residues at each position are highlighted with distinct colors. The sequences are arranged according to their phylogenetic clustering patterns.

Supplementary Figure 2 Integrated analysis of *Ipomoea GATA* genes. **(A)** Maximum Likelihood phylogenetic tree of *Ipomoea* GATA proteins, classified into four evolutionarily distinct subfamilies (I-IV). **(B)** Distribution of 20 conserved protein motifs identified by MEME suite, with color-coded annotation. Protein length scale provided. **(C)** Gene structure organization with exons (orange boxes), untranslated region (green boxes) and introns (black lines), scaled by the bottom ruler.

Supplementary Figure 3 *Cis*-acting elements prediction in the *Ipomoea GATA* gene promoters. **(A)** Phylogenetic tree. **(B)***Cis*-regulatory elements distribution. The 1500 bp upstream promoter regions of the *Ipomoea GATA* genes were subjected to *cis*-element analysis using PlantCARE software.
