# Supplementary figures and images for: Comparative analysis of the GATA transcription factors in seven Ipomoea species
Source: Front Plant Sci. 2025 Nov 19;16:1714791. doi: 10.3389/fpls.2025.1714791 (PMC12672914; doi:10.3389/fpls.2025.1714791)

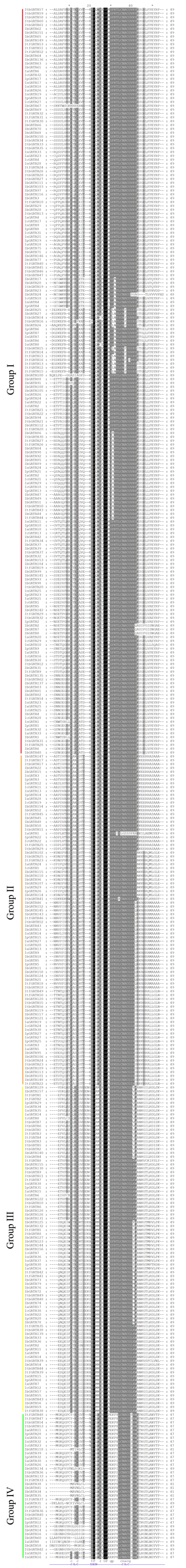

Supplement: Supplementary Figure 2 — Integrated analysis of Ipomoea GATA genes. (A) Maximum Likelihood phylogenetic tree of Ipomoea GATA proteins, classified into four evolutionarily distinct subfamilies (I-IV). (B) Distribution of 20 conserved protein motifs identified by MEME suite, with color-coded annotation. Protein length scale provided. (C) Gene structure organization with exons (orange boxes), untranslated region (green boxes) and introns (black lines), scaled by the bottom ruler. [file Image2.jpeg]
